# Neuroanatomical changes in early Parkinson’s disease with mild cognitive impairment: gray matter and white matter damage

**DOI:** 10.3389/fneur.2025.1641820

**Published:** 2025-09-17

**Authors:** Yaping Niu, Changlian Tan, Qin Shen, Sainan Cai, Qinru Liu, Min Wang, Congli Huang, Yiran Lin, Sinan Deng, Haiyan Liao

**Affiliations:** ^1^Department of Radiology, The Second Xiangya Hospital, Central South University, Changsha, China; ^2^Clinical Research Center for Medical Imaging in Hunan Province, Changsha, China

**Keywords:** early Parkinson’s disease, mild cognitive impairment, surface-based morphometry, voxel-based morphometry, tract-based spatial statistics

## Abstract

**Objectives:**

This study aims to investigate gray matter (GM) and white matter (WM) changes in early Parkinson’s disease (PD) patients with mild cognitive impairment (PD-MCI) using high-resolution T1-weighted and diffusion-weighted MR images.

**Methods:**

We recruited 40 PD-MCI patients, 26 PD patients with normal cognition (PD-NC), and 40 healthy controls (HC). Voxel-based morphometry (VBM) and surface-based morphometry (SBM) were performed to assess the relationship between gray matter volume, cortical thickness, and cognitive ability. Microstructural white matter changes were evaluated using tract-based spatial statistics (TBSS) with diffusion tensor imaging measures.

**Results:**

White matter structural abnormalities were widespread in PD-MCI patients (corpus callosum, corona radiata, superior longitudinal fasciculus, left cerebral peduncle, and left corticospinal tract), with more pronounced involvement in the left cerebral hemisphere compared to healthy controls. Additionally, PD-MCI patients exhibited localized cortical atrophy in the left parieto-occipital region (calcarine, lingual gyrus, and precuneus), left parahippocampal gyrus, fusiform gyrus and entorhinal cortex. A significant positive correlation was observed between reduced gray matter volume (GMV) in the left parieto-occipital region and lower MoCA scores in the PD-MCI group (*p* < 0.001, *R* = 0.565).

**Conclusion:**

Even in early-stage disease, our study demonstrates widespread WM microstructural damage but only subtle GM atrophy in PD-MCI, particularly in the left hemisphere. These findings provide new evidence to enhance our understanding of the pathogenic mechanisms and pathological basis underlying cognitive impairment in Parkinson’s disease.

## 1 Introduction

Cognitive impairment is one of the more common non-motor symptoms of Parkinson’s disease (PD), which can appear in the early stages of PD, even earlier than the onset of motor symptoms (1). Cognitive impairment associated with PD can be divided into subjective cognitive decline, mild cognitive impairment (PD-MCI), and PD dementia (PDD) according to its severity. Studies have shown that about 25% of PD patients have mild cognitive impairment at the time of early diagnosis (2), and up to 83% of patients develop dementia after 20 years (3). The occurrence of PDD increases the disability rate and mortality rate of PD patients, seriously affects patients’s quality of life, and is a risk factor for early death. Therefore, early identification of PD-MCI crucial for preventing or delaying its progression to PDD. At present, the diagnosis of PD-MCI mainly relies on neuropsychological assessments. Early clinical diagnosis is difficult, and its specific mechanisms remain unclear, with a lack of objective indicators for early diagnosis.

In recent years, with the development of neuroimaging technologies and the widespread application of various magnetic resonance imaging techniques, early diagnosis of PD-MCI has become possible. Numerous studies have identified both structural brain changes and functional abnormalities in PD-MCI patients. Previous Voxel-based morphometry (VBM) studies have indicated a significant association between cognitive decline and gray matter atrophy in PD patients. Patients with PD-MCI show reduced gray matter volume (GMV) in the frontal, parietal, and temporal lobes, and even involving limbic system regions (including the amygdala, hippocampus, and cingulate gyrus) and the cerebellum (4, 5). Surface-based morphometry (SBM) studies associated with Parkinson’s disease-related cognitive impairment have also indicated that the progression of cognitive impairment is associated with degeneration in cortical regions of the bilateral frontal and temporoparietal areas (6). In diffusion imaging studies, some research has shown that PD-MCI exhibits an extensive pattern of white matter (WM) abnormalities (7–9), and WM diffusion changes may contribute to predict cognitive decline (9, 10). Moreover, studies have reported abnormal synchronization of neuronal activity and functional connectivity in multiple brain regions in PD-MCI patients, including the parietal lobe, temporal lobe (including the fusiform gyrus), and insula (11, 12). These findings collectively suggest that patients with PD-MCI exhibit widespread structural brain changes and functional disturbances, which may serve as important indicators for the early identification of PD-MCI patients.

VBM enables automatic measurement of brain structure and morphology, and is an imaging analysis technique for objectively detecting changes in gray matter volume. However, in the early stages of cognitive impairment, SBM may be more sensitive than VBM in identifying cortical structural changes related to PD (13). Moreover, the brain exists in three-dimensional space and has folds, therefore VBM alone is insufficient to detect early cortical changes in PD, as it can only detect voxels with low sensitivity to overlapping areas under specific predictive effects (14). SBM analysis provides more detailed structural information of the brain using structural MRI (sMRI) data, including local anatomical changes and microstructural features of the cortical surface. VBM focuses on overall structural information of brain regions, while SBM focuses on analyzing cortical morphological features. Therefore, combining various parameters from both VBM and SBM can enable a more comprehensive and systematic study of changes in gray matter structure, which helps reveal pathological features of the disease from a neuroanatomical perspective.

Diffusion-weighted imaging (DWI) is a non-invasive and effective method for capturing microstructural properties of white matter in the brain. It detects microstructural changes in tissues by utilizing the anisotropic diffusion of water molecules in biological tissues. Diffusion tensor imaging (DTI), a common model applied to DWI data, provides quantitative metrics such as fractional anisotropy (FA) and mean diffusivity (MD). FA reflects the overall directionality of water diffusion, with values ranging from 0 (isotropic) to 1 (highly anisotropic). Higher FA values indicate more organized structures like white matter (WM) tracts, while lower values are typical of gray matter (GM) or cerebrospinal fluid (15). FA is highly sensitive to changes in axonal diameter, density, and myelin integrity (16), and has been shown to detect microstructural brain changes in Parkinson’s disease, with lower FA values significantly correlated with greater disease severity (17). MD measures the overall diffusion rate, with increased MD often indicating pathological changes such as edema or tissue loss (18). Axial Diffusivity (AD) reflects diffusion along fiber tracts and is linked to axonal integrity, while Radial Diffusivity (RD) represents diffusion across fibers and relates to myelin health (18). Together, these DTI parameters (FA, MD, AD, RD) provide a more complete view of white matter microstructure. Tract-based spatial statistics (TBSS), based on fiber tract tracing, skeletonizes the white matter fibers to ensure that each voxel on the FA skeleton map represents the FA value at the center of the nearest white matter fiber tract. This improves the accuracy of image smoothing and registration in previous DTI techniques, making it more sensitive to microstructural changes in white matter. White matter structural changes can be detected even before gray matter changes are observed through SBM or VBM (19). Furthermore, it has been hypothesized that TBSS might provide higher sensitivity than SBM or VBM in identifying early microstructural changes in PD-MCI patients. However, there have been relatively few TBSS studies on cognitive impairment in PD, and existing studies have small sample sizes (20). Its clinical efficacy as a sensitive biomarker for cognitive impairment in PD patients still needs further validation.

The current research on structural brain changes in PD-MCI is limited, and no consensus has been reached so far. Filippi et al. (21) reported that patients who converted from PD to PD-MCI showed cortical atrophy in the parietal and occipital lobes, while those who converted from PD to PDD exhibited additional involvement of the frontal and temporal lobes, Zhou et al. (22) found that PD-MCI converters had early atrophy in the right temporal lobe and progressive atrophy in the frontal lobe. However, some researchers did not find any gray matter atrophy in PD-MCI (23). This inconsistency may be attributed to several factors, including differences in sample size, severity of cognitive impairment, variability of the techniques used, and differences in the criteria used to assess cognitive impairment. In addition, previous studies mainly focused on analyzing GMV or cortical thickness as a single indicator, and rarely conducted joint analyses of gray and white matter structural damage. It has been reported that in the early stages of the disease, widespread changes in the microstructure of white matter have already occurred in the absence of gray matter atrophy and cognitive impairment (19). Structural damage to white matter may lead to trans-synaptic axonal degeneration and neuronal degeneration, which in turn affect the microstructure of gray matter (19). Given the hypothesis that white matter damage precedes neuronal loss in the corresponding gray matter regions, investigating microstructural alterations in both gray and white matter during the early stages of Parkinson’s disease may provide deeper insights into the underlying mechanisms of disease progression.

This study employs a combination of TBSS, VBM, and SBM analysis methods to conduct a comprehensive assessment of whole-brain gray and white matter microstructural alterations in PD-MCI in the early stages of Parkinson’s disease. This multifaceted approach examines the sensitivity of these methods, with the aim of enhancing early identification of mild cognitive impairment in Parkinson’s disease, identifying imaging biomarkers for PD-related mild cognitive impairment, and exploring potential neuropathological mechanisms underlying the progression of cognitive decline in Parkinson’s disease.

## 2 Materials and methods

### 2.1 Study subjects

This study was approved by the Medical Ethics Committee of the Second Xiangya Hospital of Central South University. Written informed consent was obtained from all participants. Early-stage patients with primary PD patients who attended our Hospital between December 2020 and November 2024 were recruited, including 41 males and 25 females, aged 38–78 years, with a mean age of 57.30 ± 11.44 years.

**Figure 1:**
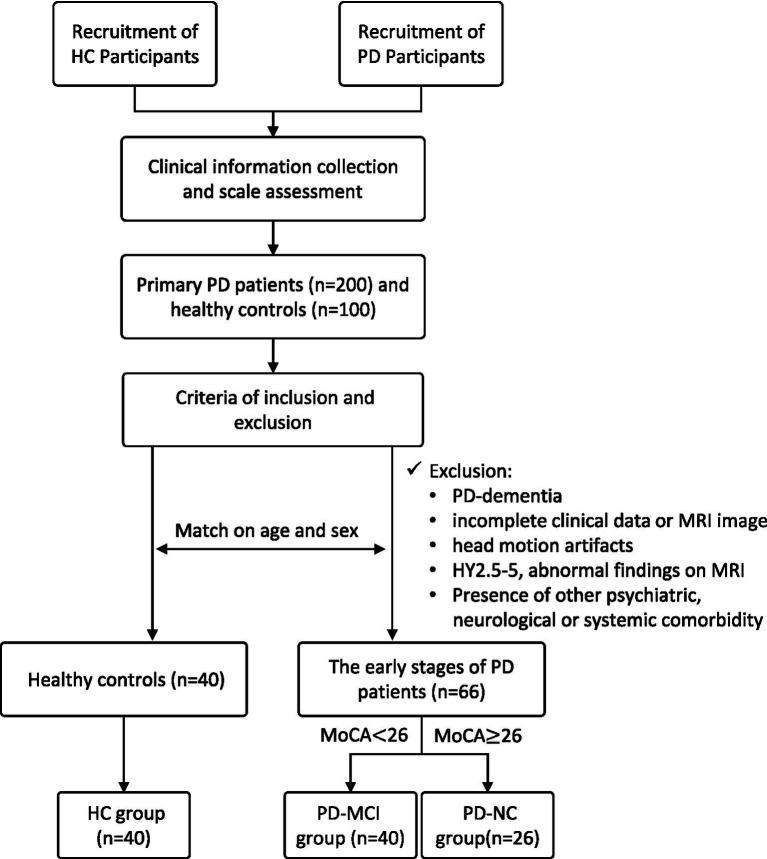
Flowchart of participants inclusion and exclusion criteria.

According to the Level I diagnostic criteria for PD-MCI proposed by the Movement Disorder Society (MDS) in May 2012 (24), which simplified the classification without specifying PD-MCI subtypes. PD-MCI is defined as subjective or objective cognitive impairment with a MoCA score below 26 and no impairment in activities of daily living (ADL). PD-CN is defined as a MoCA score ≥26 and no ADL impairment (25). The enrolled early-stage PD patients (Hoehn and Yahr stage 1–2.5) were classified into two groups: those with mild cognitive impairment (PD-MCI group, *n* = 40) and those with normal cognition (PD-NC group, *n* = 26) (Figure 1).

The inclusion criteria for PD were as follows: (1) met the 2015 MDS clinical diagnostic criteria for PD and Hoehn and Yahr stage 1–2.5; (2) had not received antiparkinsonian medication or had withdrawn from such treatment for more than 12 h; and (3) were right-handed.

Exclusion criteria for PD included: (1) presence of dementia, as indicated by Mini-Mental State Examination (MMSE) scores below 17 for illiterate individuals, below 20 for those with 1–6 years of education, and below 23 for those with 7 or more years of education; (2) evidence of organic brain lesions or a history of neurological or psychiatric disorders; and (3) inability to complete MRI scanning or contraindications to MRI.

In addition, 40 healthy controls (HC group), matched for age and sex with the PD participants, were recruited, comprising 18 males and 22 females, aged 42–79 years, with a mean age of 54.98 ± 7.53 years.

The inclusion criteria for HC were as follows: (1) matched in terms of age, gender with PD patients; (2) right-handedness.

The exclusion criteria for HC were as follows: (1) presence of significant structural brain lesions or other neurologic or psychiatric diseases; (2) presence of cognitive impairment based on MMSE or MoCA scores.

Before MRI scanning, all PD participants underwent the following assessments: the Unified Parkinson’s Disease Rating Scale (UPDRS), Hoehn and Yahr staging (HY), Mini-Mental State Examination (MMSE), Montreal Cognitive Assessment (MoCA), and Hamilton Depression Rating Scale (HAMD).

### 2.2 MRI acquisition

All imaging was performed using a 3.0 T Signa Premier MRI scanner (GE Healthcare, United States). Participants were instructed to lie supine with their heads positioned first into the scanner bore. Foam padding was placed around the head to minimize motion, and earplugs were provided to reduce scanner noise.

Initially, all subjects underwent conventional MRI sequences (T1WI, T2WI, and T2-FLAIR) to screen for overt structural brain abnormalities. Subsequently, high-resolution three-dimensional T1-weighted magnetization-prepared rapid gradient echo (3D-MPRAGE) images and diffusion tensor imaging (DTI) data were acquired.

3D-MPRAGE parameters: Number of layers (Sagittal) = 176, slice thickness = 1.0 mm with no inter-slice gap, repetition time (TR) = 1,900 ms, echo time (TE) = 2.01 ms, flip angle = 9°, field of view (FOV) = 256 × 256 mm^2^.

DTI parameters: At each location, 64 diffusion-weighted directions with b = 1,000 s/mm^2^ plus 1 b0 images per individual were acquired with the following parameters: TR = 6,400 ms, TE = 86 ms, FOV = 256 × 256 mm^2^, matrix = 128 × 128, voxel size = 2 × 2 × 2.5 mm^3^, slice thickness = 2.5 mm with no inter-slice gap.

### 2.3 Data processing and statistical analysis

#### 2.3.1 Tract-based spatial statistics

Diffusion tensor imaging (DTI) data were processed using the FMRIB Software Library (FSL).^1^ DTI data were preprocessed using FSL software, including motion correction, eddy current distortion correction, and brain extraction to remove non-brain tissues. Subsequently, individual FA, MD, AD, and RD maps were generated based on the preprocessed data. Tract-Based Spatial Statistics (TBSS) was subsequently performed to analyze group differences.

The analysis followed standard TBSS procedures: the FMRIB58_FA_1mm template was opened as the standard space, and all individual FA maps were registered to this template. Non-linear registration was conducted, followed by the creation of a mean FA image and a mean FA skeleton. The mean FA skeleton was thresholded at FA > 0.2 to exclude peripheral tracts and non-white matter regions, resulting in a skeletonized representation of the major white matter pathways. Each participant’s aligned FA data were projected onto this skeleton to obtain subject-specific skeletonized FA maps. Next, the same procedures were applied to create mean templates and corresponding skeleton maps for MD, AD, and RD.

TBSS statistical analyses were conducted using gender, age and education years as covariates. A general linear model (GLM) design matrix was constructed using FSL, and two-sample *t*-tests were performed between each pair of the three groups (PD-MCI, PD-NC, and HC) for FA, MD, RD, and AD maps using the FSL randomize program with 5,000 permutations, generating Family-wise Error (FWE) corrected statistical maps. A result was considered statistically significant when the corrected *p* < 0.05 and the number of contiguous voxels in the cluster exceeded 30. For intergroup comparisons, anatomical localization of significant white matter regions was determined using the Johns Hopkins University (JHU) ICBM-DTI-81 white matter atlas as a reference. For the white matter regions showing statistically significant differences based on TBSS analysis, further correlation analyses were performed using Spearman correlation analysis to assess the associations between these diffusion metrics and Montreal Cognitive Assessment (MoCA) scores.

#### 2.3.2 Surface-based morphometry and voxel-based morphometry

All original MRI images in DICOM format were converted to the standard NIfTI format using the dcm2niix medical image conversion tool. Image preprocessing was conducted using the Computational Anatomy Toolbox (CAT12)^2^ implemented in SPM12 (Wellcome Centre for Human Neuroimaging, London, United Kingdom) running on the MATLAB 2013b platform.

For voxel-based morphometry (VBM) analysis, preprocessing included tissue segmentation using Tissue Probability Maps (TPMs), followed by spatial normalization to the standard Montreal Neurological Institute (MNI) space using the DARTEL algorithm. This yielded modulated normalized gray matter (GM), white matter (WM), and cerebrospinal fluid (CSF) volume maps for each subject.

For surface-based morphometry (SBM) analysis, preprocessing included segmentation, surface reconstruction, topological correction, spherical registration, and spatial normalization (26), Central surfaces and cortical thickness maps were generated for both cerebral hemispheres. Image quality was assessed using both visual inspection and the automated quality control module in CAT12, with all included images achieving a quality rating of grade B or higher.

Using the “Surface Tools” module in CAT12, additional surface-based morphological parameters were derived from the central surfaces, including the gyrification index (GI), fractal dimension (FD), and sulcal depth. Smoothing was applied to all morphological maps: GM volume maps were smoothed with an 8-mm full-width at half maximum (FWHM) Gaussian kernel, while cortical thickness, GI, FD, and sulcal depth maps were smoothed using a 20-mm FWHM Gaussian kernel.

Statistical analyses of smoothed GMV, cortical thickness, GI, FD, and sulcal depth were performed using one-way analysis of variance (ANOVA) in SPM12/CAT12. Age, sex, and years of education were included as covariates in all models. Additionally, total intracranial volume (TIV) was included as a covariate in the GMV analysis. Multiple comparisons were corrected using the family-wise error (FWE) rate, and statistically significant clusters were identified. *Post hoc* tests were conducted on extracted values from significant clusters. Finally, Spearman correlation analysis was used to assess the relationships between MoCA scores and morphological indices (GMV, cortical thickness, GI, FD, sulcal depth), with significance set at two-tailed *p* < 0.05.

#### 2.3.3 Statistical analysis of clinical data

All statistical analyses were performed using SPSS version 25.0. Data with normal distributions are presented as mean ± standard deviation ( $\bar{x}$ ±s), while non-normally distributed data are reported as median and interquartile range (lower quartile and upper quartile). One-way analysis of variance (ANOVA) was used to compare age and years of education among the three groups. The chi-square ( $x^{2}$ ) test was used to assess differences in sex distribution. Independent-samples t-test was employed to compare UPDRS-III scores between the PD-MCI and PD-NC groups. The Kruskal–Wallis H test and Mann–Whitney U test were used to evaluate between-group differences (either among all three groups or pairwise comparisons) in disease duration, total UPDRS scores, Hoehn and Yahr (HY) stage, and MMSE scores. Bonferroni correction was applied for multiple comparisons, with a corrected significance threshold of *p* < 0.017 (0.05/3).

## 3 Results

### 3.1 Demographic and clinical characteristics

The demographic and clinical characteristics of all participants are summarized in Table 1. There were no significant differences among the PD-MCI, PD-NC, and HC groups in terms of age or sex distribution, indicating successful group matching on these variables. No significant differences were observed between the PD-MCI and PD-NC groups with respect to disease duration, modified Hoehn and Yahr (H-Y) staging, UPDRS-III scores, or HAMD scores.

**Table 1:** The demographic and clinical data of all participants.

Variable	PD-MCI (*N* = 40)	PD-NC (*N* = 26)	HC (*N* = 40)	*p*-value
Age	58.40 ± 9.14	54.57 ± 9.96	54.98 ± 7.53	0.169ᵇ
Sex (M/F)	27/13	14/12	18/22	0.126ᵃ
Duration (yrs)	1.75 (0.56, 3.00)	1.50 (1.00, 3.00)	NA	0.847ᶜ
Education (yrs)	7.33 ± 3.77	10.73 ± 3.32	8.46 ± 3.60	0.001ᵇ
MMSE	26.50 (25.00, 27.75)	29.00 (29.00, 30.00)	29.00 (26.25, 30.00)	<0.001ᵉ
H-Y stage	2.00 (1.13, 2.50)	1.75 (1.50, 2.13)	NA	0.294ᶜ
UPDRS-III	20.85 ± 14.49	17.80 ± 9.58	NA	0.349ᵈ
HAMD	4.00 (2.25, 6.75)	4.50 (1.50, 10.00)	NA	0.621ᶜ
MoCA	21.00 (16.25, 23.00)	27.00 (26.75, 29.00)	NA	0.000^c^
Side (R/L)	16/14	11/15	NA	0.410ᵃ

Data are presented as mean ± standard deviation, median (lower quartile and upper quartile) for continuous variables, or frequencies for categorical ones. For comparisons of demographics: ^a^*p*-value for the gender difference was obtained by chi-square test. ^b^*p*-values were obtained by one-way analysis of variance (ANOVA) tests. ^c^*p*-value was obtained by Mann–Whitney U test. ^d^*p*-values were obtained by two-sample *t*-test. ^e^*p*-values were obtained by Kruskal–Wallis H test. PD-MCI, Parkinson’s disease with mild cognitive impairment; PD-NC, Parkinson’s disease with normal cognition; HC, healthy controls; MMSE, Mini-Mental State Examination; H-Y, Hoehn-Yahr stage; UPDRS-III, Unified Parkinson’s Disease Rating Scale motor section; HAMD, Hamilton Depression Rating Scale; MoCA, Montreal Cognitive Assessment; NA, not applicable.

However, significant group differences were found in years of education (*p* = 0.001), as well as MMSE and MoCA scores (*p* < 0.001). Specifically, the PD-MCI group exhibited lower education levels and MoCA scores compared to the PD-NC group (*p* < 0.001). Furthermore, MMSE scores in the PD-MCI group were significantly lower than those in both the PD-NC and HC groups (*p* < 0.001).

### 3.2 Group comparisons and correlation analysis of white matter damage based on TBSS

**Figure 2:**
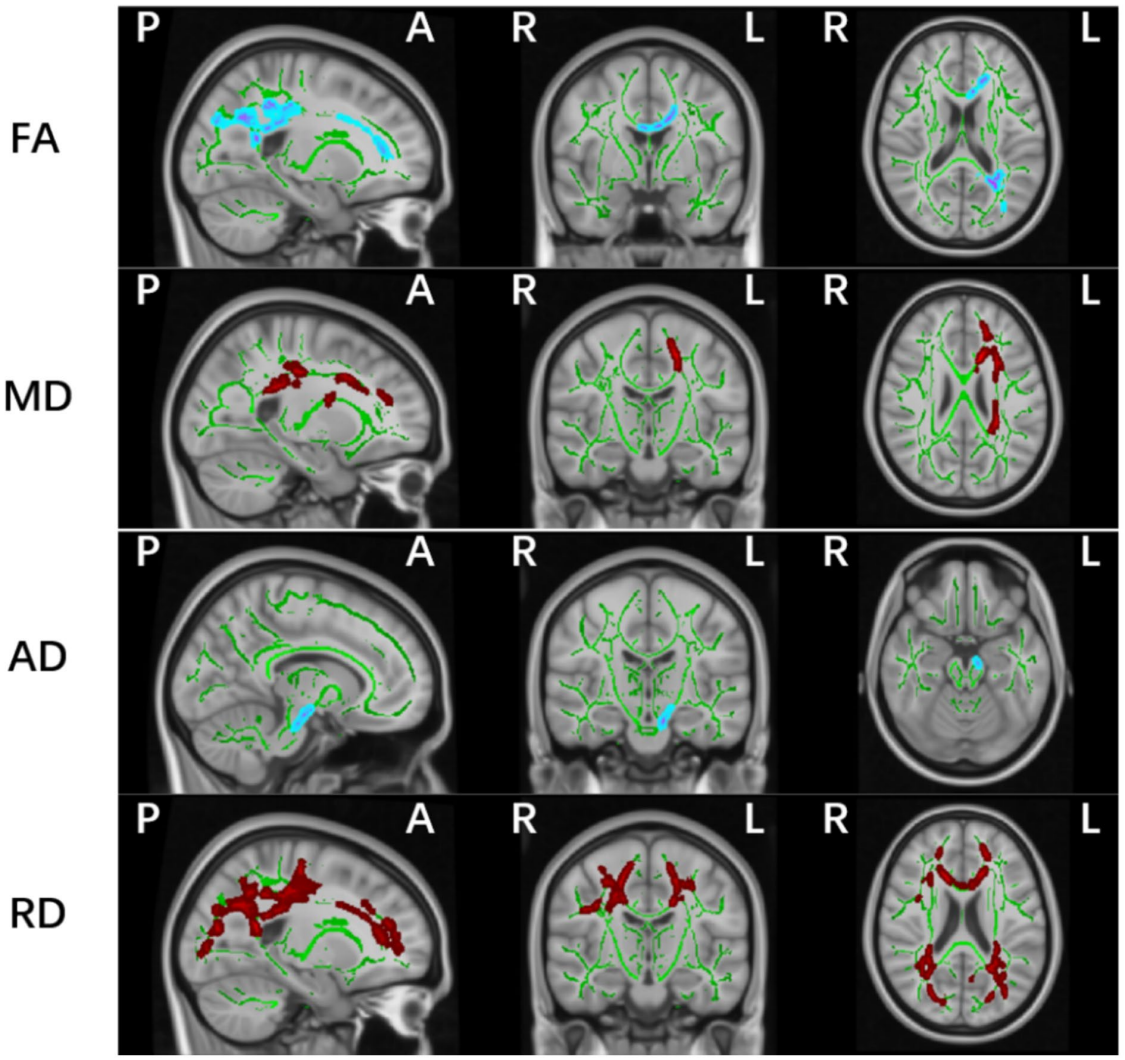
Significant white matter differences between PD-MCI and HC groups (*p* < 0.05, FWE controlled). FA, fractional anisotropy; AD, axial; MD, mean and RD, radial diffusion. The background image is the standard MNI152 brain template. Green voxels represent the white matter skeleton of all subjects; Red colors denote decreased and blue colors increased cortical thickness in patients compared with controls; L, left; R, right; (P), posterior; (A), anterior.

Whole-brain TBSS analysis of DTI data revealed statistically significant white matter differences in multiple parameters (FA, MD, AD, and RD) between the PD-MCI and HC groups (*p* < 0.05, TFCE corrected; Table 2 and Figure 2). The affected regions were as follows:

FA (Fractional Anisotropy):
Significant decreases were observed in the corpus callosum (including the genu, body, and splenium), the left anterior, superior, and posterior corona radiata, and the left posterior thalamic radiation (including the optic radiation).
MD (Mean Diffusivity):
Increased MD values were detected in the corpus callosum (genu and body), the left anterior, superior, and posterior corona radiata, the left retrolenticular part of internal capsule, the left posterior thalamic radiation (including the optic radiation), and the superior longitudinal fasciculus.
AD (Axial Diffusivity):
Significant differences were identified in the left corticospinal tract and cerebral peduncle.
RD (Radial Diffusivity):
Affected regions included the corpus callosum (genu, body, splenium), bilateral anterior, superior, and posterior corona radiata, bilateral superior longitudinal fasciculus, posterior thalamic radiation (including the optic radiation), the right sagittal stratum (including the inferior longitudinal fasciculus and inferior fronto-occipital fasciculus), and the left retrolenticular part of internal capsule.

**Table 2:** Significant clusters based on TBSS parameters between PD-MCI and HC groups.

Metric	Cluster	Anatomical region	MNI coordinates, mm			*p*-value	Cluster sizes
			X	Y	Z		
FA	1	CC (genu and body), Left CR, Left PTR	−19	37	10	0.029	3,113
MD	2	CC (genu and body), Left CR, Left PTR, Left SLF, Left RLIC	−25	−37	26	0.046	1,882
AD	1	Left CP	−9	−21	−25	0.048	34
	2	Left CST, Left CP	−13	−14	−12	0.047	62
RD	2	CC (genu), Left PCR	−33	−68	24	0.043	1,669
	3	CC, CR, SLF, PTR, Right ILF and IFO, Left RLIC	−14	20	24	0.024	12,991

Tract-based spatial statistics (TBSS) analysis. FA, fractional anisotropy; AD, axial; MD, mean and RD, radial diffusion; CC, corpus callosum; CR, anterior corona radiata; PCR, posterior corona radiata; SLF, superior longitudinal fasciculus; PTR, posterior thalamic radiation; RLIC, retrolenticular part of internal capsule; CP, cerebral peduncle; CP, cerebral peduncle; CST, corticospinal tract; IFO, Inferior fronto-occipital fasciculus; ILF, inferior longitudinal fasciculus; MNI, Montreal Neurologic Institute.

No significant differences were observed in FA, MD, AD, or RD values between the PD-NC and HC groups, or between the PD-MCI and PD-NC groups.

Spearman correlation analysis between the mean values of altered AD regions in the PD-MCI group and their MoCA scores revealed a positive correlation in the left corticospinal tract and cerebral peduncle (*p* < 0.05, *R* = 0.277).

### 3.3 Group comparisons and correlation analysis of gray matter volume based on VBM

**Figure 3:**
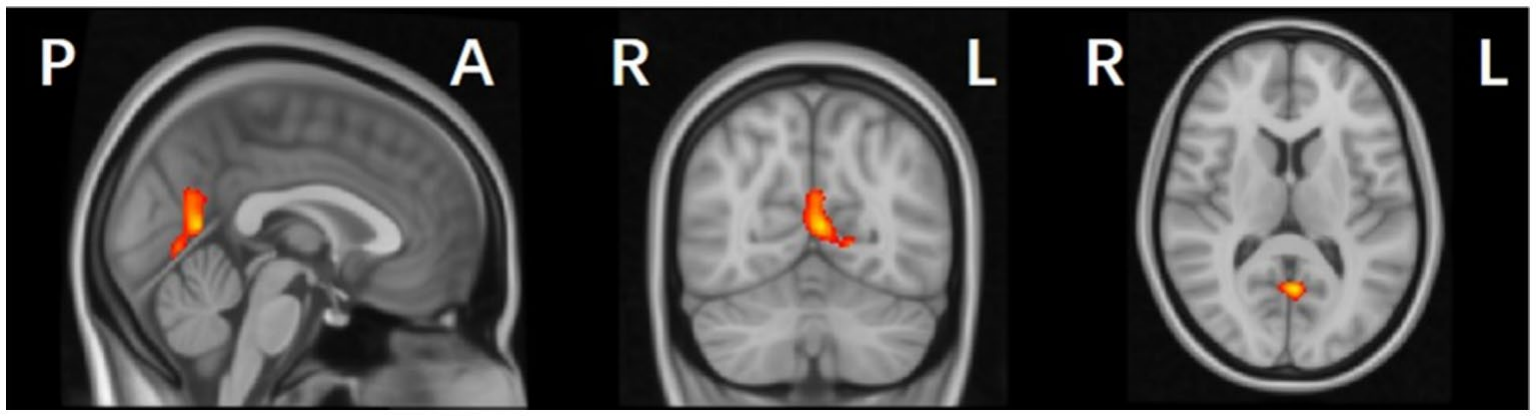
Gray matter volume (GMV) reduction regions in PD-MCI compared with HC group (*p* < 0.05, FWE corrected).

Compared with the HC and PD-NC groups, the PD-MCI group exhibited localized reductions in gray matter volume (GMV) in the left parieto-occipital region, including the calcarine, lingual gyrus, and precuneus (*p* < 0.05, FWE corrected; Table 3 and Figure 3). No regions with significantly increased GMV were found in the PD-MCI group. There were no statistically significant GMV differences between the PD-NC and HC groups.

**Table 3:** Brain regions with reduced gray matter volume in the PD-MCI group.

Anatomic region	Cluster sizes	*p*-value	MNI coordinates		
			X	Y	Z
Left calcarine *	241	0.046	−1.5	−61.5	10.5
Left lingual gyrus	305				
Left precuneus	145				

VBM, voxel-based morphometry; PD-MCI, Parkinson’s disease with mild cognitive impairment; MNI, Montreal Neurologic Institute; *represents the peak points. And the X, Y, and Z coordinates of MNI represent the position of peak points.

**Figure 4:**
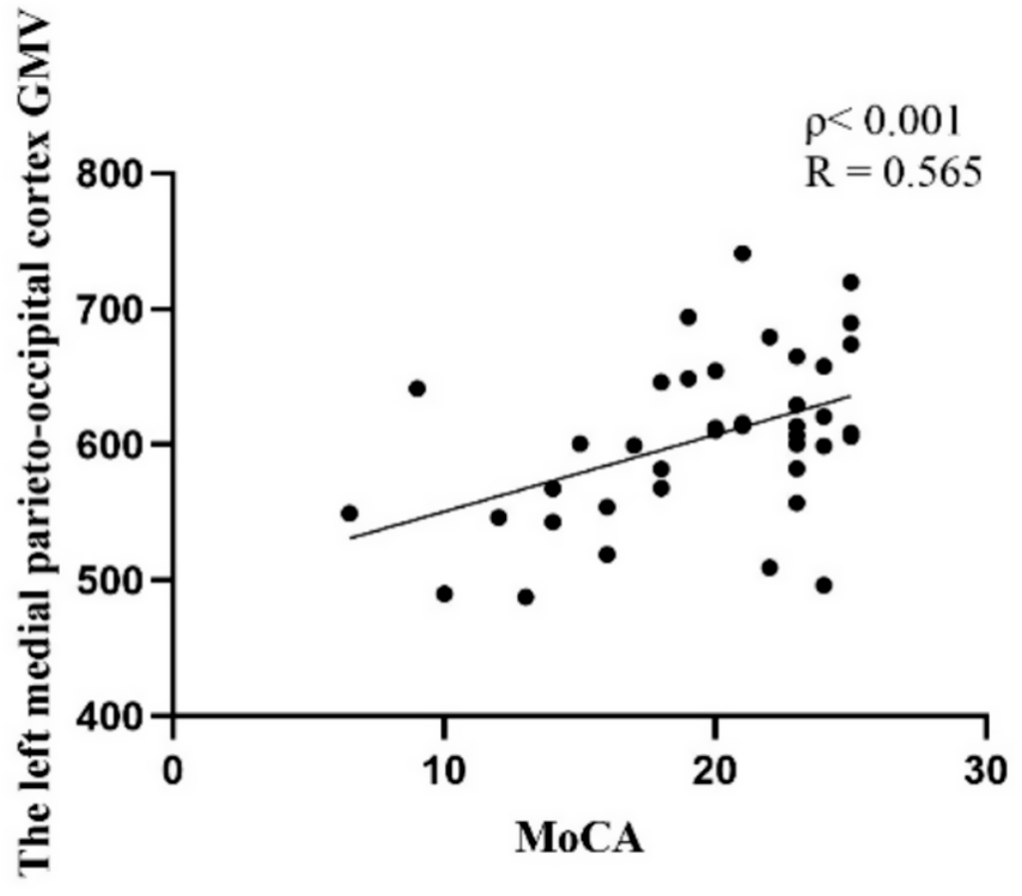
A plot for the significant correlation analysis within PD-MCI patients, GMV reductions in the left medial parieto-occipital cortex were positively correlated with MoCA scores (*p* < 0.001, *R* = 0.565).

Table 3 and Figure 3 illustrate the cortical regions with GMV reductions in the PD-MCI group. Spearman correlation analysis showed that GMV reductions in the left medial parieto-occipital cortex were positively correlated with MoCA scores (*p* < 0.001, *R* = 0.565) (Figure 4), suggesting that structural atrophy in this region may be associated with cognitive decline in early-stage Parkinson’s disease.

### 3.4 Cortical thickness and cortical complexity group comparisons and correlation analysis based on surface-based morphometry

**Figure 5:**
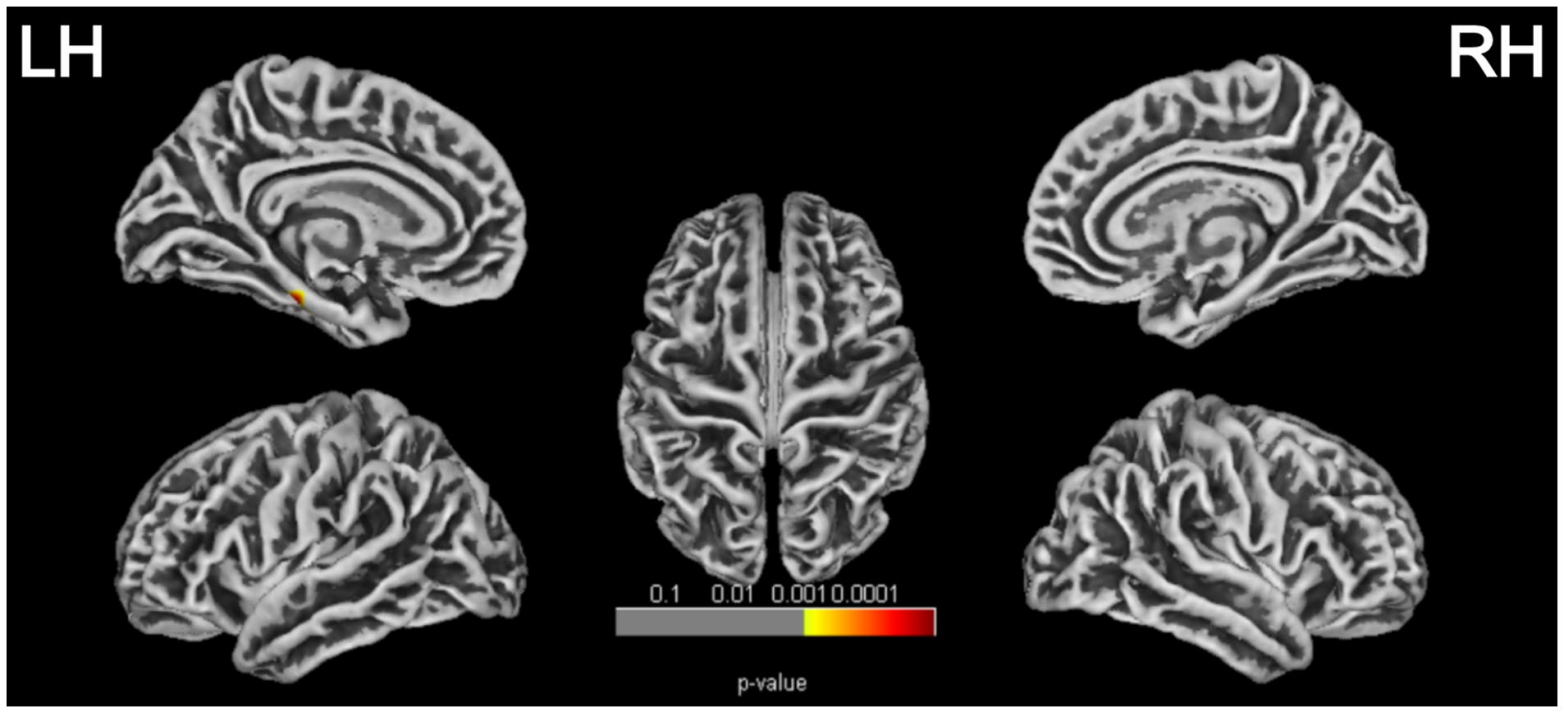
Cortical thickness reduction regions in patients with PD-MCI (*p* < 0.05, FWE corrected). LH, left hemisphere; RH, right hemisphere.

SBM analysis revealed a cortical thickness (CT) reduction region in the left cerebral hemisphere comprising 325 vertices (*p* = 0.00002, FWE corrected; Table 4 and Figure 5), involving the following brain areas: the left parahippocampus (53%), fusiform gyrus (35%), and entorhinal cortex (12%). *Post hoc* analysis showed that the CT value in the PD-MCI group was significantly lower compared with both the PD-NC group (*p* = 0.004, Bonferroni corrected) and the HC group (*p* < 0.001, Bonferroni corrected). There were no statistically significant group differences in fractal dimension (FD), gyrification index (GI), or sulcal depth (SD). No significant correlation was found between the reduced CT region in the left hemisphere of the PD-MCI group and MoCA scores (*p* > 0.05).

**Table 4:** Cortical thickness reduction regions in patients with PD-MCI (*p* < 0.05, FWE corrected).

Anatomic region	Cluster sizes	*F*-value	MNI coordinates		
			X	Y	Z
Parahippocampal (53%)	658	0.00002	−21	−27	−23
Fusiform gyrus (35%)					
Entorhinal cortex (12%)					

## 4 Discussion

This study employed a combination of tract-based spatial statistics (TBSS), voxel-based morphometry (VBM), and surface-based morphometry (SBM) to systematically investigate alterations in whole-brain gray and white matter structures in early-stage PD patients with mild cognitive impairment (PD-MCI). The aim was to identify potential neuroimaging biomarkers associated with early cognitive decline in PD. Our findings revealed widespread microstructural disruptions in white matter across both cerebral hemispheres in the PD-MCI group, with a predominance in the left hemisphere. These alterations likely reflect compromised white matter integrity, including axonal degeneration and demyelination. Moreover, regional gray matter atrophy was localized to the medial occipitoparietal cortex of the left hemisphere, encompassing the pericalcarine cortex, lingual gyrus, and precuneus. Surface-based analysis further identified cortical thinning in the left parahippocampal gyrus, entorhinal cortex, and fusiform gyrus. Together, these structural abnormalities may underlie the cognitive deficits observed in early PD and highlight candidate imaging biomarkers for the early detection of cognitive impairment.

Our study reveals that even at early stages of cognitive decline, PD patients may exhibit widespread white matter (WM) alterations, particularly in the corpus callosum, corona radiata, and superior longitudinal fasciculus (SLF), consistent with previous findings (7, 9). These results support the hypothesis that cognitive impairment in PD is associated with disruptions in widespread cognitive-related fiber tracts, particularly those subserving attention, visuospatial processing, and executive function. The corpus callosum, the largest commissural fiber bundle in the brain, facilitates interhemispheric transmission of visual and somatosensory information across the frontal, parietal, and occipital cortices. It is critically involved in memory, attention, motor coordination, and sensory integration (27, 28). Fibers in the splenium connect the medial occipitoparietal cortices of both hemispheres, which are crucial for processing speed and executive control (29). Bledsoe et al. used DTI to characterize WM microstructure in the corpus callosum and found that impaired interhemispheric and callosal-cortical connectivity may contribute to cognitive dysfunction in PD (30). The corona radiata plays a central role in mediating communication and coordination between different cortical regions. Previous studies have linked alterations in the anterior, superior, and posterior corona radiata with impairments in attention, executive function, and visuospatial abilities (27). Similarly, the superior longitudinal fasciculus (SLF), a major association fiber tract connecting the frontal, parietal, and temporal cortices, is known to support a broad range of cognitive processes, including visual attention, language, and complex reasoning (31–34).

It is well established that the neuropathological hallmarks of PD include the accumulation of *α*-synuclein-containing Lewy bodies and the degeneration of dopaminergic neurons. Pathological evidence suggests that misfolded α-synuclein can propagate along axonal pathways, potentially explaining WM alterations as a result of trans-synaptic spread of pathology from the brainstem to the telencephalon and neocortex (35). WM microstructural changes may thus serve as indicators of the pathological progression of PD and could potentially predict cognitive decline. However, further studies with larger samples are required to validate these findings. Importantly, our study revealed that axial diffusivity (AD) reductions in the left cerebral peduncle and corticospinal tract were positively correlated with MoCA scores in PD-MCI patients, suggesting a link between microstructural integrity and global cognitive performance. The corticospinal tract (CST) is responsible for conveying motor signals from the primary motor cortex to the spinal cord and plays a critical role in voluntary motor control. Recent evidence suggests that cortical projections from somatosensory, cingulate, and insular regions also contribute to the CST (36). Emerging studies highlight that the basal ganglia are intricately connected with cortical regions through parallel cortico-basal ganglia-thalamo-cortical loops (37), allowing for integration of sensorimotor, associative, and limbic information. This interconnectivity implies that damage to either motor or cognitive circuits could result in impairments across both domains (38). Future research should investigate how corticospinal tract abnormalities may contribute to the co-occurrence of cognitive and motor dysfunction in PD, particularly in the context of PD-related cognitive impairment (PD-CI).

In contrast to the widespread white matter alterations, our VBM and SBM analyses revealed focal cortical structural abnormalities in PD-MCI patients, mainly in the temporal and parieto-occipital regions. These included gray matter volume (GMV) reduction in the left medial parieto-occipital cortex (involving the pericalcarine cortex, lingual gyrus, and precuneus), and cortical thinning in the left parahippocampal gyrus, fusiform gyrus, and entorhinal cortex-regions that may serve as potential imaging biomarkers for cognitive decline in early PD.

Recent studies have consistently reported associations between cortical atrophy in the frontal, temporal, and parieto-occipital regions and cognitive impairment in PD (4, 39, 40). However, we did not observe significant frontal thinning in our cohort. This may reflect differences in disease stage, as our participants were at an earlier phase of PD. Furthermore, variability in individual disease trajectories, diagnostic criteria, imaging methods, and sample sizes may account for this discrepancy. Longitudinal studies have shown that PD patients who later converted to MCI (PD-NC to MCI) exhibited patterns of cortical alterations involving posterior brain regions (such as the parietal and occipital cortices), compared to healthy controls and PD-NC, indicating that these regions may be the earliest sites affected during the progression of cognitive decline (21). Our results further support this notion. Recent studies in early-diagnosed PD patients have suggested that posterior cortical atrophy in the early stages of PD is associated with reduced striatal dopamine transporter uptake, which may help explain the posterior pattern of cortical atrophy observed in our study. Moreover, we found a strong correlation between reduced GMV in the left medial parieto-occipital cortex and lower MoCA scores in PD-MCI patients. This provides further evidence for the neuropathological basis of cognitive impairment and may aid in disease severity prediction. We also observed thinning of the left fusiform gyrus, involved in visual cognition such as face recognition (41). A similar finding was reported in a study on drug-naive early-stage PD-MCI patients (42).

Interestingly, our findings revealed that white matter tract damage in PD-MCI patients was predominantly localized in the left cerebral hemisphere. In addition, structural abnormalities were observed in the left medial parieto-occipital cortex, as well as in the left parahippocampal gyrus, entorhinal cortex, and fusiform gyrus. This study further extends and supports the conclusion proposed by Claassen et al. that the left hemisphere may exhibit increased vulnerability in early-stage PD. One possible explanation is that the left nigrostriatal system may be more susceptible to early neurodegeneration compared to the right side (43). Similar patterns of left-lateralized early neuropathological changes have also been described in other neurodegenerative diseases, including Alzheimer’s disease (44) and frontotemporal dementia (45). Moreover, neurodegeneration in PD may be influenced by both handedness and asymmetrical dopaminergic depletion. In our study, all participants were right-handed. Parkinson’s disease is known to involve asymmetric degeneration of dopaminergic neurons in the nigrostriatal pathway, and evidence from DAT-SPECT studies has shown that a significantly greater proportion of right-handed PD patients exhibit predominant dopaminergic loss in the left hemisphere (46). This suggests that the interaction between hemispheric dominance and asymmetrical neurodegeneration may render the dominant (typically left) hemisphere more vulnerable to early pathological changes.

Our results showed no significant cortical structural differences between the PD-NC group and healthy controls, which is consistent with several previous studies (47, 48). However, other research has reported reduced cortical thickness in PD-NC patients compared to healthy controls (5). Several factors may account for these inconsistent findings in the literature, including sample size, heterogeneity within PD-NC cohorts—particularly with respect to disease stage—as well as methodological variability in techniques used to assess gray matter changes. For instance, studies including more advanced PD patients may fail to capture early anatomical alterations that occur at the onset of the disease. Moreover, we did not detect significant differences in gray matter volume or cortical thickness between the PD-MCI and PD-NC groups. Notably, several prior studies have also reported subtle or inconsistent structural differences between these groups (21, 49), possibly due to heterogeneity in disease stage, cognitive profiles, and sample sizes. These findings suggest that while structural MRI may detect changes in PD-MCI, such alterations can be subtle, and larger or longitudinal studies may be necessary to identify consistent group-level effects. Additionally, one possible reason for the absence of significant structural differences in our study may be the inclusion of age, sex, and years of education as covariates in our statistical model. Previous studies have demonstrated that both age and educational attainment influence cognitive impairment and cortical atrophy in PD (50, 51), which may have reduced between-group variance and contributed to the lack of significant findings in our analysis.

This study has several limitations. An important limitation is the use of level I PD-MCI criteria instead of level II criteria. Although the level II criteria provide a more comprehensive assessment, they can be challenging to apply in routine clinical practice for every patient with PD. This limitation may have contributed to potential misclassification or under-detection of cognitive changes. Another important limitation concerns the sensitivity of the structural imaging methods employed. Both SBM and VBM are primarily designed to detect macroscopic gray matter atrophy, and may lack sensitivity to subtle or early microstructural changes. This limitation could partly explain the relatively sparse and localized cortical changes observed in the present study. Neurite Orientation Dispersion and Density Imaging (NODDI) have shown greater sensitivity to microstructural degeneration in both white and gray matter and may provide complementary insights in future investigations. Additionally, as a cross-sectional study, it cannot provide insights into longitudinal changes. Future longitudinal studies are needed to validate our findings. Despite these limitations, our findings suggest that alterations in cortical thickness, gray matter volume, and white matter microstructure in key brain regions may serve as useful MRI-based anatomical markers for identifying patients with mild cognitive impairment in Parkinson’s disease.

## 5 Conclusion

In summary, even at an early stage of the disease, patients with Parkinson’s disease and mild cognitive impairment (PD-MCI) exhibit widespread white matter microstructural abnormalities and localized cortical atrophy in the left parietal-occipital cortex, parahippocampal gyrus, entorhinal cortex, and fusiform gyrus. These changes may be closely associated with the pathogenesis of cognitive impairment in PD. Additionally, the lateralized nature of white matter damage—predominantly affecting the left hemisphere—further supports the hypothesis of left-hemispheric vulnerability in early-stage PD. These structural alterations provide morphological evidence for understanding the pathophysiological mechanisms underlying cognitive decline in PD and hold promise as non-invasive neuroimaging biomarkers for identifying and monitoring PD-related cognitive impairment.

## Data Availability

The original contributions presented in the study are included in the article/supplementary material, further inquiries can be directed to the corresponding author.

## Footnotes

^1^ https://fsl.fmrib.ox.ac.uk

^2^ http://www.neuro.uni-jena.de/cat/

## References

[1] Weintraub D Burn DJ. Parkinson’s disease: the quintessential neuropsychiatric disorder. Mov Disord. (2011) 26:1022–31. doi: 10.1002/mds.23664, PMID: 21626547 PMC3513835

[2] Pedersen KF Larsen JP Tysnes O-B Alves G. Natural course of mild cognitive impairment in Parkinson disease: a 5-year population-based study. Neurology. (2017) 88:767–74. doi: 10.1212/Wnl.0000000000003634, PMID: 28108638

[3] Aarsland D Andersen K Larsen JP Lolk A. Prevalence and characteristics of dementia in Parkinson disease: an 8-year prospective study. Arch Neurol. (2003) 60:387. doi: 10.1001/archneur.60.3.387, PMID: 12633150

[4] Mihaescu AS Masellis M Graff-Guerrero A Kim J Criaud M Cho SS. Brain degeneration in Parkinson’s disease patients with cognitive decline: a coordinate-based meta-analysis. Brain Imaging Behav. (2019) 13:1021–34. doi: 10.1007/s11682-018-9922-0, PMID: 29971686

[5] Gao Y Nie K Huang B Mei M Guo M Xie S. Changes of brain structure in Parkinson’s disease patients with mild cognitive impairment analyzed via VBM technology. Neurosci Lett. (2017) 658:121–32. doi: 10.1016/j.neulet.2017.08.028, PMID: 28823894

[6] Gasca-Salas C García-Lorenzo D Garcia-Garcia D Clavero P Obeso JA Lehericy S. Parkinson’s disease with mild cognitive impairment: severe cortical thinning antedates dementia. Brain Imaging Behav. (2019) 13:180–8. doi: 10.1007/s11682-017-9751-6, PMID: 28710667

[7] Wang W Mei M Gao Y Huang B Qiu Y Zhang Y. Changes of brain structural network connection in Parkinson’s disease patients with mild cognitive dysfunction: a study based on diffusion tensor imaging. J Neurol. (2020) 267:933–43. doi: 10.1007/s00415-019-09645-x, PMID: 31792673

[8] Inguanzo A Segura B Sala-Llonch R Monte-Rubio G Abos A Campabadal A. Impaired structural connectivity in Parkinson’s disease patients with mild cognitive impairment: a study based on probabilistic tractography. Brain Connect. (2021) 11:380–92. doi: 10.1089/brain.2020.0939, PMID: 33626962 PMC8215419

[9] Minett T Su L Mak E Williams G Firbank M Lawson RA. Longitudinal diffusion tensor imaging changes in early Parkinson’s disease: icicle-pd study. J Neurol. (2018) 265:1528–39. doi: 10.1007/s00415-018-8873-0, PMID: 29696499

[10] Chondrogiorgi M Astrakas LG Zikou AK Weis L Xydis VG Antonini A. Multifocal alterations of white matter accompany the transition from normal cognition to dementia in Parkinson’s disease patients. Brain Imaging Behav. (2019) 13:232–40. doi: 10.1007/s11682-018-9863-7, PMID: 29629498

[11] Li M Liu T Zhang T Li MG Liu TF Zhang TH. Alterations of regional homogeneity in Parkinson’s disease with mild cognitive impairment: a preliminary resting-state fMRI study. Neuroradiology. (2020) 62:327–34. doi: 10.1007/s00234-019-02333-7, PMID: 31822931

[12] Zhu Y Yang B Zhou C Gao C Hu Y Yin WF. Cortical atrophy is associated with cognitive impairment in Parkinson’s disease: a combined analysis of cortical thickness and functional connectivity. Brain Imaging Behav. (2022) 16:2586–600. doi: 10.1007/s11682-022-00714-w, PMID: 36044168

[13] Pereira JB Ibarretxe-Bilbao N Marti M Compta Y Junqué C Bargallo N. Assessment of cortical degeneration in patients with Parkinson’s disease by voxel-based morphometry, cortical folding, and cortical thickness. Hum Brain Mapp. (2012) 33:2521–34. doi: 10.1002/hbm.21378, PMID: 21898679 PMC6870035

[14] Xu L Groth KM Pearlson G Schretlen DJ Calhoun VD. Source-based morphometry: the use of independent component analysis to identify gray matter differences with application to schizophrenia. Hum Brain Mapp. (2009) 30:711–24. doi: 10.1002/hbm.20540, PMID: 18266214 PMC2751641

[15] Schulte T Sullivan EV Müller-Oehring EM Adalsteinsson E Pfefferbaum A. Corpus callosal microstructural integrity influences interhemispheric processing: a diffusion tensor imaging study. Cereb Cortex. (2005) 15:1384–92. doi: 10.1093/cercor/bhi020, PMID: 15635059

[16] Smith SM Jenkinson M Johansen-Berg H Rueckert D Nichols TE Mackay CE. Tract-based spatial statistics: voxelwise analysis of multi-subject diffusion data. Neuroimage. (2006) 31:1487–505. doi: 10.1016/j.neuroimage.2006.02.024, PMID: 16624579

[17] Loane C Politis M Kefalopoulou Z Valle-Guzman N Paul G Widner H. Aberrant Nigral diffusion in Parkinson’s disease: a longitudinal diffusion tensor imaging study: abnormal diffusion in PD. Mov Disord. (2016) 31:1020–6. doi: 10.1002/mds.26606, PMID: 27104232

[18] Li Z Liu W Xiao C Wang X Zhang X Yu M. Abnormal white matter microstructures in Parkinson’s disease and comorbid depression: a whole-brain diffusion tensor imaging study. Neurosci Lett. (2020) 735:135238. doi: 10.1016/j.neulet.2020.135238, PMID: 32645398

[19] Rektor I Svátková A Vojtíšek L Zikmundová I Vaníček J Király A. White matter alterations in Parkinson’s disease with normal cognition precede grey matter atrophy. PLoS One. (2018) 13:e0187939. doi: 10.1371/journal.pone.0187939, PMID: 29304183 PMC5755732

[20] Chen F Wu T Luo Y Li Z Guan Q Meng X. Amnestic mild cognitive impairment in Parkinson’s disease: white matter structural changes and mechanisms. PLoS One. (2019) 14:e0226175. doi: 10.1371/journal.pone.0226175, PMID: 31830080 PMC6907797

[21] Filippi M Canu E Donzuso G Stojkovic T Basaia S Stankovic I. Tracking cortical changes throughout cognitive decline in Parkinson’s disease. Mov Disord. (2020) 35:1987–98. doi: 10.1002/mds.28228, PMID: 32886420

[22] Zhou C Guan X Guo T Guan XJ Zeng QL Gao T. Progressive brain atrophy in Parkinson’s disease patients who convert to mild cognitive impairment. CNS Neurosci Ther. (2020) 26:117–25. doi: 10.1111/cns.13188, PMID: 31278861 PMC6930819

[23] Amboni M Tessitore A Esposito F Santangelo G Picillo M Vitale C. Resting-state functional connectivity associated with mild cognitive impairment in Parkinson’s disease. J Neurol. (2015) 262:425–34. doi: 10.1007/s00415-014-7591-5, PMID: 25428532

[24] Litvan I Goldman JG Tröster AI Schmand BA Weintraub D Petersen RC. Diagnostic criteria for mild cognitive impairment in Parkinson’s disease: Movement Disorder Society task force guidelines. Mov Disord. (2012) 27:349–56. doi: 10.1002/mds.24893, PMID: 22275317 PMC3641655

[25] Kim JI Sunwoo MK Sohn YH Lee PH Hong JY. The MMSE and MOCA for screening cognitive impairment in less educated patients with Parkinson’s disease. J Mov Disord. (2016) 9:152–9. doi: 10.14802/jmd.16020 27667187 PMC5035941

[26] Dahnke R Yotter RA Gaser C. Cortical thickness and central surface estimation. NeuroImage. (2013) 65:336–48. doi: 10.1016/j.neuroimage.2012.09.050, PMID: 23041529

[27] Zheng Z Shemmassian S Wijekoon C Kim W Bookheimer SY Pouratian N. Dti correlates of distinct cognitive impairments in Parkinson’s disease. Hum Brain Mapp. (2014) 35:1325–33. doi: 10.1002/hbm.22256, PMID: 23417856 PMC3664116

[28] Theilmann RJ Reed JD Song DD Huang MX Lee RR Litvan I. White-matter changes correlate with cognitive functioning in Parkinson’s disease. Front Neurol. (2013) 4:37. doi: 10.3389/fneur.2013.00037 23630517 PMC3624087

[29] Huang L Chen X Sun W Chen H Ye Q Yang D. Early segmental white matter fascicle microstructural damage predicts the corresponding cognitive domain impairment in cerebral small vessel disease patients by automated fiber quantification. Front Aging Neurosci. (2021) 12:598242. doi: 10.3389/fnagi.2020.598242, PMID: 33505302 PMC7829360

[30] Bledsoe IO Stebbins GT Merkitch D Goldman JG. White matter abnormalities in the corpus callosum with cognitive impairment in Parkinson disease. Neurology. (2018) 91:e2244–55. doi: 10.1212/Wnl.0000000000006646 30429273 PMC6329325

[31] Ribeiro M Yordanova YN Noblet V Herbet G Ricard D. White matter tracts and executive functions: a review of causal and correlation evidence. Brain. (2024) 147:352–71. doi: 10.1093/brain/awad308, PMID: 37703295

[32] Cochereau J Lemaitre A-L Wager M Moritz-Gasser S Duffau H Herbet G. Network-behavior mapping of lasting executive impairments after low-grade glioma surgery. Brain Struct Funct. (2020) 225:2415–29. doi: 10.1007/s00429-020-02131-5, PMID: 32813155

[33] Herbet G Duffau H. Contribution of the medial eye field network to the voluntary deployment of visuospatial attention. Nat Commun. (2022) 13:328. doi: 10.1038/s41467-022-28030-3, PMID: 35039507 PMC8763913

[34] Parlatini V Radua J Dell’acqua F Leslie A Simmons A Murphy DG. Functional segregation and integration within fronto-parietal networks. NeuroImage. (2017) 146:367–75. doi: 10.1016/j.neuroimage.2016.08.031, PMID: 27639357 PMC5312783

[35] Sarasso E Agosta F Piramide N Filippi M. Progression of grey and white matter brain damage in Parkinson’s disease: a critical review of structural MRI literature. J Neurol. (2021) 268:3144–79. doi: 10.1007/s00415-020-09863-8, PMID: 32378035

[36] Galea MP Darian-Smith I. Multiple corticospinal neuron populations in the macaque monkey are specified by their unique cortical origins, spinal terminations, and connections. Cereb Cortex. (1994) 4:166–94. doi: 10.1093/cercor/4.2.166, PMID: 8038567

[37] Moustafa AA Chakravarthy S Phillips JR Crouse JJ Gupta A Frank MJ. Interrelations between cognitive dysfunction and motor symptoms of Parkinson’s disease: behavioral and neural studies. Rev Neurosci. (2016) 27:535–48. doi: 10.1515/revneuro-2015-0070 26982614

[38] Mcgregor MM Nelson AB. Circuit mechanisms of Parkinson’s disease. Neuron. (2019) 101:1042–56. doi: 10.1016/j.neuron.2019.03.004, PMID: 30897356

[39] Gorges M Kunz MS Müller H Liepelt‐Scarfone I Storch A Dodel R. Longitudinal brain atrophy distribution in advanced Parkinson’s disease: what makes the difference in “cognitive status” converters? Hum Brain Mapp. (2020) 41:1416–34. doi: 10.1002/hbm.24884, PMID: 31789477 PMC7267933

[40] Sampedro F Martínez-Horta S Marín-Lahoz J Pagonabarraga J Kulisevsky J. Longitudinal intracortical diffusivity changes in de-novo Parkinson’s disease: a promising imaging biomarker. Parkinsonism Relat Disord. (2019) 68:22–5. doi: 10.1016/j.parkreldis.2019.09.031, PMID: 31621613

[41] Hauw F Sangaré A Munoz-Musat E Meyniel C Di Donato N Chokron S. Are we aware of neural activity in primary visual cortex? A neuropsychological case study. Ann Clin Transl Neurol. (2024) 11:1365–70. doi: 10.1002/acn3.52038, PMID: 38509632 PMC11093234

[42] Jia X Wang Z Yang T Li Y Gao S Wu G. Entorhinal cortex atrophy in early, drug-naive Parkinson’s disease with mild cognitive impairment. Aging Dis. (2019) 10:1221. doi: 10.14336/ad.2018.1116, PMID: 31788334 PMC6844592

[43] Claassen DO Mcdonell KE Donahue M Rawal S Wylie SA Neimat JS. Cortical asymmetry in Parkinson’s disease: early susceptibility of the left hemisphere. Brain Behav. (2016) 6:e00573. doi: 10.1002/brb3.573, PMID: 28031997 PMC5167000

[44] Donix M Burggren AC Scharf M Marschner K Suthana NA Siddarth P. APOE associated hemispheric asymmetry of entorhinal cortical thickness in aging and Alzheimer’s disease. Psychiatry Res Neuroimaging. (2013) 214:212–20. doi: 10.1016/j.pscychresns.2013.09.006, PMID: 24080518 PMC3851589

[45] Rohrer JD Clarkson MJ Kittus R Rossor MN Ourselin S Warren JD. Rates of hemispheric and lobar atrophy in the language variants of frontotemporal lobar degeneration. J Alzheimer's Dis. (2012) 30:407–11. doi: 10.3233/Jad-2012-111556, PMID: 22406442 PMC4606976

[46] Fiorenzato E Antonini A Bisiacchi P Weis L Biundo R. Asymmetric dopamine transporter loss affects cognitive and motor progression in Parkinson’s disease. Mov Disord. (2021) 36:2303–13. doi: 10.1002/mds.28682, PMID: 34124799 PMC8596815

[47] Mak E Su L Williams GB Firbank MJ Lawson RA Yarnall AJ. Baseline and longitudinal grey matter changes in newly diagnosed Parkinson’s disease: icicle-pd study. Brain. (2015) 138:2974–86. doi: 10.1093/brain/awv211, PMID: 26173861 PMC4671477

[48] Pereira JB Svenningsson P Weintraub D Brønnick K Lebedev A Westman E. Initial cognitive decline is associated with cortical thinning in early Parkinson disease. Neurology. (2014) 82:2017–25. doi: 10.1212/Wnl.0000000000000483, PMID: 24808018 PMC4105257

[49] Hanganu A Bedetti C Degroot C Mejia-Constain B Lafontaine A-L Soland V. Mild cognitive impairment is linked with faster rate of cortical thinning in patients with Parkinson’s disease longitudinally. Brain. (2014) 137:1120–9. doi: 10.1093/brain/awu036, PMID: 24613932

[50] Nie K Gao Y Mei M Guo M Huang Z Wang L. The clinical characteristics and cognitive features of mild cognitive impairment in Parkinson’s disease and the analysis of relevant factors. J Clin Neurosci. (2019) 63:142–8. doi: 10.1016/j.jocn.2019.01.021, PMID: 30732989

[51] Santos-García D de T Cores C Feal M García I Íñiguez M. Cognitive impairment and dementia in young onset Parkinson’s disease. J Neurol. (2023) 270:5793–812. doi: 10.1007/s00415-023-11921-w, PMID: 37578489

